# Food Packaging Based on Nanomaterials

**DOI:** 10.3390/nano9091224

**Published:** 2019-08-29

**Authors:** Amparo López-Rubio, Maria José Fabra, Marta Martínez-Sanz

**Affiliations:** Instituto de Agroquímica y Tecnología de los Alimentos (IATA-CSIC), Food Safety and Preservation Department, 46980 Paterna, Valencia, Spain

The use of nanomaterials for food applications is a rapidly evolving field and, given the specific properties of nanomaterials and their tremendous potential, an increased number of material innovations that contribute to improved food quality and safety are foreseen. Although the possibilities are unlimited, and nanomaterials in the food area have been applied with different purposes [1], the development of nanocomposites for food-packaging applications has been one of the most widely explored topics. In the food-packaging area, specifically for polymeric and biopolymeric materials, incorporation of well-dispersed nanoparticles (organic and inorganic) has demonstrated their ability to improve mechanical, thermal and barrier properties without affecting the optical characteristics of the materials [2]. This is probably the area within the food nanotechnology field where more research has been done, giving rise to various commercial products. Since several years ago, research efforts have been going a step further and, thus, the use of these nanoparticles is aimed, not only at improving the quality and safety of the packaged food passively, but at playing an active role in food preservation and food quality enhancement. These novel materials are known as active/bioactive packages, which are able to release/absorb certain substances to change either the inner packaging atmosphere or even the food product. The aim of combining these active/bioactive substances included in the packaging structure with nanoparticles is to modulate their release or sorption or even to use the nanoparticles as active substances themselves (such as antimicrobial packages containing nanometals). However, it is also true that very little is known about the potential migration of nanoparticles from the packages and their subsequent potential toxicity.

This special issue is devoted to compiling several papers on these fascinating topics. Specifically, five research papers and one review paper are included in the special issue “Food Packaging based on Nanomaterials”. The manuscripts by Sharif et al. [3] and Bugatti et al. [4], both make use of the electrospinning technique to generate active packaging coatings aimed at improving food quality and safety. Electrospinning has emerged as a very promising tool to generate nanomaterials for a broad number of applications, highlighting their potential in the biomedical area (given the ability of the technique to generate structures that simulate cell tissues), but which has also proven to be a useful and versatile technique for various food applications [5,6,7]. The electrospinning process uses high-voltage electric fields to produce electrically charged “jets” from viscoelastic polymer solutions which, on drying by means of the evaporation of the solvent, produce diverse ultrathin structures. In the food-packaging area it has demonstrated its ability to create polymeric/biopolymeric layers on top of a diverse range of materials, attaining excellent attachment between the layers and, thus, avoiding the need to use additional adhesive layers [8]. This opens up the possibility of generating bio-based multilayer structures for food-packaging applications and the incorporation of such thin electrospun layers has proved to result in improved properties of the materials both used as intermediate tie layers (being able to increase oxygen barrier properties) [9] or as external packaging coatings (modifying the hydrophobicity of the materials) [10]. An additional advantage of the technique is that active substances may be incorporated within these ultrathin structures to give rise to packaging coatings with functional properties. Specifically, the manuscript of Sharif et al. [3] describes the development of gliadin-based structures containing ferulic acid to generate antioxidant packaging structures. As a strategy to improve the stability of this antioxidant molecule, ferulic acid was previously incorporated within cyclodextrins to generate inclusion complexes that were subsequently electrospun using zein as the carrier biopolymer [3]. The manuscript by Bugatti et al. [4] reports on antimicrobial packaging structures using lysozyme encapsulated by electrospinning as the antimicrobial agent. In this case, halloysite nanotubes (HNTs) were used as carriers for the antimicrobial enzyme and these hybrid materials were tested on chicken slices, demonstrating a reduction in bacterial growth and, thus, potentially contributing to improving food safety [4].

Combining inherent antimicrobial polymers (like chitosan) with antimicrobial nanoparticles (like zinc oxide) was the approach used by Al-Naamani et al. [11]. These hybrid materials were applied as coatings on low density polyethylene (LDPE) films and tested for the preservation of vegetables, demonstrating their ability to retard microbial and fungal growth during storage of these fresh foods [11].

A different strategy was explored by Muriel-Galet et al. [12] in another manuscript from this Special Issue collection to develop smart packaging materials. In this case, gated mesoporous silica particles were anchored to ethylene vinyl alcohol films to generate pH-sensitive materials, able to release the encapsulated substances at a given pH value. The optimal conditions for anchoring the loaded nanoparticles were evaluated and, as a proof of concept, the release of rhodamine B in a food simulant at two different pH conditions was studied, confirming the stimuli-sensitive behavior of the developed packaging materials [12].

The toxicological aspects of packaging nanomaterials are also covered in this Special Issue through the work of Bott and Franz [13], who studied nanoparticle migration from laponite–polymer nanocomposites. It is well-known that, in order to enhance the properties of nanoclay-containing polymeric nanocomposites, the nanoclays need to be properly exfoliated and intercalated within the polymeric matrix [2]. Migration will depend not only on the size of the nanoparticle, but also can be affected by the loading. Therefore, in this work, sample films with different laponite loadings were stored at high temperature (60 °C) to accelerate release, and migration in a food simulant was studied. No migration of laponite was detected once it was incorporated into the polymer matrix using a limit of detection of 22 µg laponite per Kg food, thereby assuring the safety of this specific material for food packaging applications [13].

The Special Issue is completed with a review paper from Huang et al. [14] which compiles three years of recent research findings on new developments of nanomaterials for food packaging, including both organic and inorganic-based nanomaterials. The review includes information about the synthetic methods and related properties of the materials obtained, and the biological activity of some of them together with their food applications. It also highlights the possible mechanisms of antimicrobial activity of certain nanomaterials and their health concerns [14].

In summary, this Special Issue of Nanomaterials entitled “Food Packaging based on Nanomaterials” compiles a series of original research articles and a review paper providing new insight on the preparation and on the wealth of applications of hybrid nanomaterials for food-packaging applications. We are confident that this Special Issue will provide the reader with an overall view of some of the latest prospects in this fast-evolving and cross-disciplinary field.
